# High-precision robust monitoring of charge/discharge current over a wide dynamic range for electric vehicle batteries using diamond quantum sensors

**DOI:** 10.1038/s41598-022-18106-x

**Published:** 2022-09-06

**Authors:** Yuji Hatano, Jaewon Shin, Junya Tanigawa, Yuta Shigenobu, Akimichi Nakazono, Takeharu Sekiguchi, Shinobu Onoda, Takeshi Ohshima, Keigo Arai, Takayuki Iwasaki, Mutsuko Hatano

**Affiliations:** 1 Department of Electrical and Electronic Engineering, School of Engineering, Tokyo Institute of Technology, Tokyo, Japan; 2 Yazaki Corporation, Shizuoka, Japan; 3 National Institutes for Quantum Science and Technology, Gunma, Japan

**Keywords:** Electrical and electronic engineering, Applied optics

## Abstract

Accurate prediction of the remaining driving range of electric vehicles is difficult because the state-of-the-art sensors for measuring battery current are not accurate enough to estimate the state of charge. This is because the battery current of EVs can reach a maximum of several hundred amperes while the average current is only approximately 10 A, and ordinary sensors do not have an accuracy of several tens of milliamperes while maintaining a dynamic range of several hundred amperes. Therefore, the state of charge has to be estimated with an ambiguity of approximately 10%, which makes the battery usage inefficient. This study resolves this limitation by developing a diamond quantum sensor with an inherently wide dynamic range and high sensitivity for measuring the battery current. The design uses the differential detection of two sensors to eliminate in-vehicle common-mode environmental noise, and a mixed analog–digital control to trace the magnetic resonance microwave frequencies of the quantum sensor without deviation over a wide dynamic range. The prototype battery monitor was fabricated and tested. The battery module current was measured up to 130 A covering WLTC driving pattern, and the accuracy of the current sensor to estimate battery state of charge was analyzed to be 10 mA, which will lead to 0.2% CO_2_ reduction emitted in the 2030 WW transportation field. Moreover, an operating temperature range of − 40 to + 85 °C and a maximum current dynamic range of ± 1000 A were confirmed.

## Introduction

**Figure 1:**
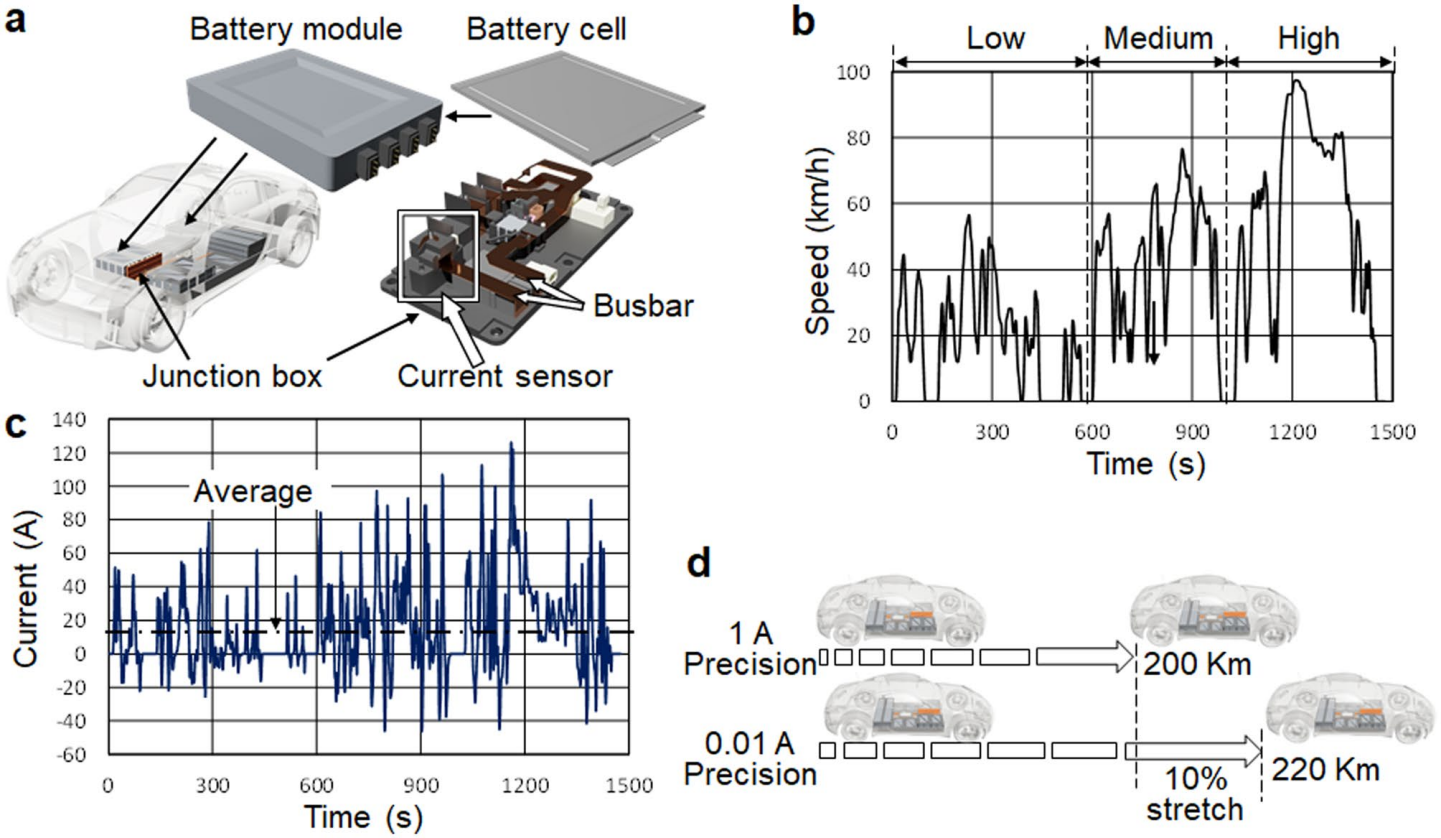
(**a**) Battery current sensor usage in the EV. Currents from the battery module with stacked battery cells pass through the busbar in the junction box and measured by the current sensor. (**b**) Driving speed (km/h) and (**c**) Converted current in the WLTC (Worldwide Harmonized Light Vehicles Test Cycle) mode^1^ assuming a typical automobile weighing 1500 kg. The maximum is 126 A and the average 14 A. (**d**) Effect of the highly accurate current sensor on stretching the EV's driving range. Estimating the battery state of charge based on the current sensor with 1 A accuracy requires 10% margin, which can be eliminated with 0.01 A accuracy sensor.

To estimate the battery state of charge in electric vehicle (EV), currently a 10% margin is necessary based on the accuracy of commercially available current sensors. The battery current sensor used in an EV is shown in Fig. 1a. Current from the battery module with stacked cells passes through the busbar in the junction box and is measured by the current sensor. Figure 1b shows the driving speed (km/h) in the Worldwide Harmonized Light Vehicles Test Cycle (WLTC) mode^1^, which is a global standard for determining the levels of pollutants, CO_2_ emissions and fuel consumption of cars and vehicles. The estimated busbar current assuming a typical automobile weighing 1500 kg is shown in Fig. 1c. The maximum current exceeds 126 A, while the average is 14 A. At present, commercially available current sensors, which can measure up to several hundred amperes, have an accuracy of 1 A. If the average current is 10 A, this accuracy requires 10% (= 1 A/10 A) margin in estimating the battery state of charge. If the accuracy can be improved to 10 mA while keeping the maximum measurable current of several hundred amperes, we can estimate the battery state of charge with 0.1% accuracy, this 10% margin becomes unnecessary and the driving range can be stretched by 10%, as shown in Fig. 1d. To determine accurately the total charge as the integral of the current value, absolute accuracy in mA is required in the current sensor. Furthermore, this measurement must be performed in the automotive temperature range of − 40 to 85 °C.

This requirement can be met by the diamond nitrogen vacancy (NV) center which offers high sensitivity from sub-nT to pT^2–14^, a wide operating temperature range based on the wide band gap of carbon crystals^15^, a wide dynamic range^16^ by the independence of the gyromagnetic ratio on the resonance frequency and the intrinsic unsaturation, and the ability to eliminate the effect of temperature drift from the magnetic field measurement by monitoring plural resonance frequencies^17–22^.

Although several diamond sensors with a sensitivity of sub-nT sufficient to detect 10 mA, and compactness for automotive equipment have been reported^11,14^, there is an unsolved need for a device that can detect 10 mA while accepting several hundred amperes, and also achieve this accuracy in a noisy automobile environment. For these purposes, this study introduces differential detection using two diamond sensors on both sides of the busbar and mixed analog–digital control for the microwave generator frequency to trace the resonance frequency over a wide dynamic range.

The remainder of this paper is organized as follows. After introducing the configuration of the busbar current differential detection system, the noise spectrum without busbar current is shown to confirm the effect of common-mode noise elimination in the frequency domain. Then, a current pulse train from an external current source with the amplitude of 100 to 1 mA is supplied to the busbar to confirm that 10 mA can be detected in the time domain in a noisy environment. Thereafter, analog–digital control of the microwave generator frequency for wide dynamic range is explained, and the busbar current from the battery module up to 130 A is evaluated as for its linearity and fluctuation to clarify the accuracy of the sensor. WLTC driving current pattern from the battery module is also confirmed to be traced in real time. Eventually, operation up to ± 1000 A from an external current source and in the temperature range of − 40 to 85 °C is indicated to discuss the vehicular application.

## Busbar current differential detection system

Differential detection using diamond sensors has been reported^23–26^ and should be particularly effective for busbar current measurements. By placing two sensors on both sides of the busbar rather than close to each other, the current magnetic field is obtained as the differential output of both sensors, and the environmental magnetic field noise is eliminated as a common mode. Furthermore, the metal busbar effectively blocks the microwave and fluorescence interference between the sensors, which ensures the accuracy of the differential detection.

**Figure 2:**
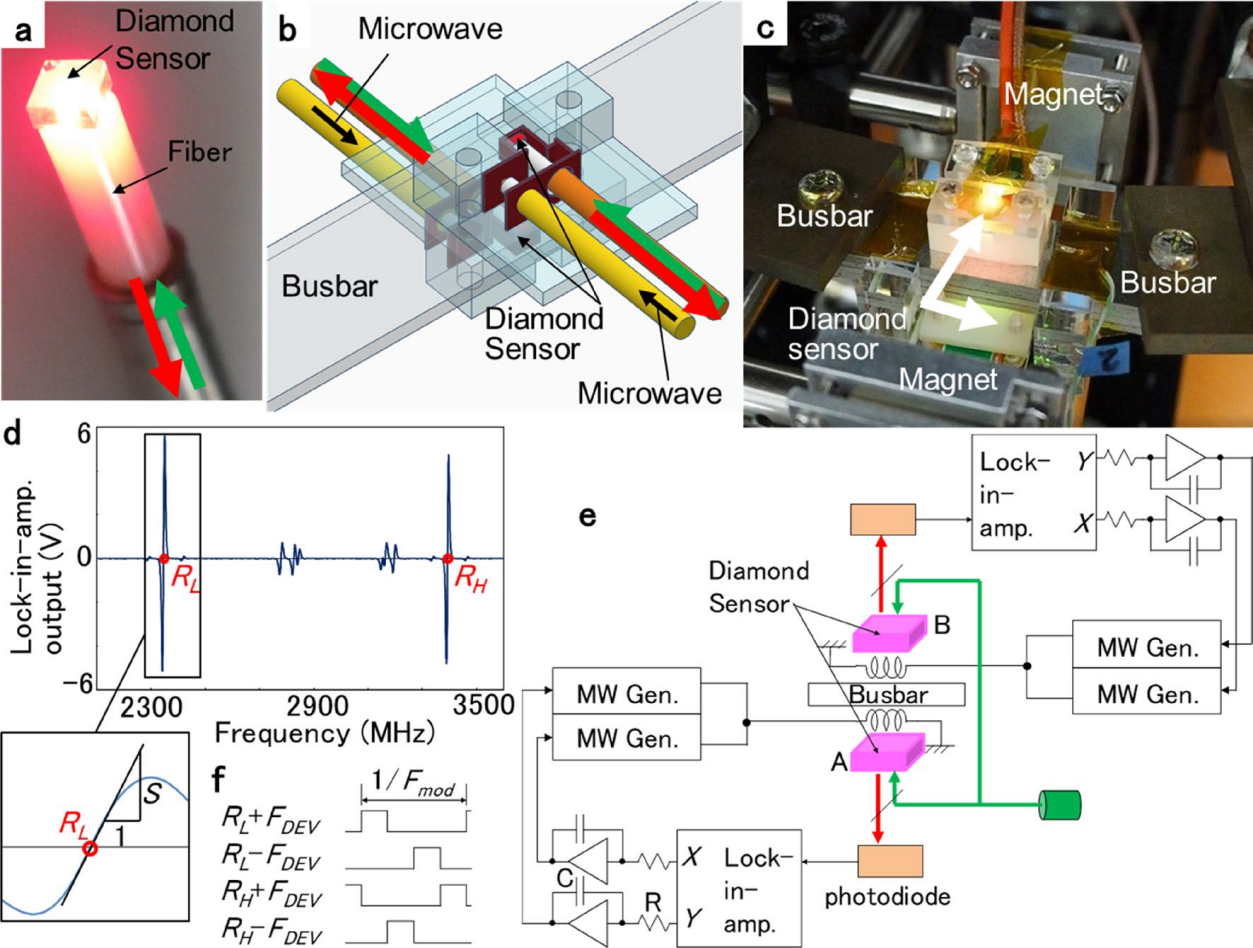
Prototype busbar current differential detection system. (**a**) 2 × 2 × 1 mm^3^ diamond sensor adhered to one end of a fiber. (**b**) Sensors A and B placed on both sides of the busbar for differential detection. (**c**) Photograph of sensors and busbar placed between a pair of magnets. (**d**) ODMR spectrum of the diamond sensor with the resonance frequency difference of the [111] NV^−^, *R*_*H*_ − *R*_*L*_ = 1050 MHz. *S* is illustrated as the slope of the ODMR at *R*_*L*_ and *R*_*H*_. (**e**) Block diagram of the busbar current measurement system. The lock-in-amplifier output difference *Y*–*X* is integrated to control the two microwave generator frequencies to trace the low and high resonance frequency. (**f**) Time division microwave frequency modulation for a lock-in-amplifier to reflect the magnetic field as its differential output. Four frequencies *R*_*L*_ ± *F*_*DEV*_ and *R*_*H*_ ± *F*_*DEV*_ generated by ± *F*_*DEV*_ are provided within one cycle of *F*_*MOD*_ without overlapping.

The prototype busbar current differential detection system is shown in Fig. 2. The sensor head consisted of a 2 × 2 × 1 mm^3^ diamond sensor adhered to one end of the fiber, as shown in Fig. 2a. Sensors A and B were placed on both sides of the busbar, as shown in Fig. 2b. The busbar current was supplied from either the battery module or the external current source, as shown in Fig. 2c. With this configuration:

$$ {\text{Sensor A senses }}\left( { - \, \left( {\text{magnetic field by the busbar current}} \right) \, + \, \left( {\text{external magnetic field}} \right)} \right) $$

$$ {\text{Sensor B senses }}\left( { + \, \left( {\text{magnetic field by the busbar current}} \right) \, + \, \left( {\text{external magnetic field}} \right)} \right) $$

Then, as the above difference, (magnetic field by the busbar current) can be obtained excluding the external magnetic field as common mode noise.

Denoting the lower and higher resonance frequencies of the negatively charged [111] NV (NV^−^) as *R*_*L*_ and *R*_*H*_, and denoting the resonance frequency difference as *RFD* ≡ *R*_*H*_ − *R*_*L*_, in the optically detected magnetic resonance (ODMR) spectrum of the diamond sensor shown in Fig. 2d, *RFD* was 1050 MHz = 19 mT × 2*γ*, where *γ* is the gyromagnetic ratio (28 MHz/mT). The 19 mT static magnetic field was provided by the Neodymium magnet.

The block diagram of the busbar current measurement system is shown in Fig. 2e. Denoting the normal and orthogonal phase outputs of the lock-in-amplifier as *X* and *Y*, and denoting their differential output as *LOD* ≡ *Y* − *X*, *LOD* equals (magnetic field) × 2*γS*, where *S* is the slope of the ODMR at the resonance frequency *R*_*L*_ and *R*_*H*_, as illustrated in Fig. 2d.

Simultaneous FM modulation is performed for the two microwave frequencies of *R*_*L*_ and *R*_*H*_. One cycle of FM modulation is divided into four periods of 90° each, and the two FM modulated microwave frequencies of *R*_*L*_ and *R*_*H*_ are supplied to the diamond sensor in a shared timing. As shown in Fig. 2f, the four frequencies *R*_*L*_ + *F*_*DEV*_, *R*_*H*_ − *F*_*DEV*_, *R*_*L*_ − *F*_*DEV*_, and *R*_*H*_ + *F*_*DEV*_ are supplied in this order without overlap. As a result, the PD output difference between *R*_*L*_ + *F*_*DEV*_ and *R*_*L*_ − *F*_*DEV*_ frequencies appears in the normal phase *X* of the lock-in amplifier. At the same time, the PD output difference between *R*_*H*_ + *F*_*DEV*_ and *R*_*H*_ − *F*_*DEV*_ frequencies appears in the orthogonal phase *Y*. The *X* and *Y* outputs of the single lock-in amplifier obtained in this way are integrated and fed back to the microwave oscillators generating *R*_*L*_ and *R*_*H*_. This allows the frequencies of the two microwave generators to trace *R*_*L*_ and *R*_*H*_ exactly. Due to this control, the frequency components of the magnetic field slower than 1/CR appear in *RFD* and those faster than 1/CR remain in *LOD*.

## Differential detection effect in the noise spectrum

**Figure 3:**
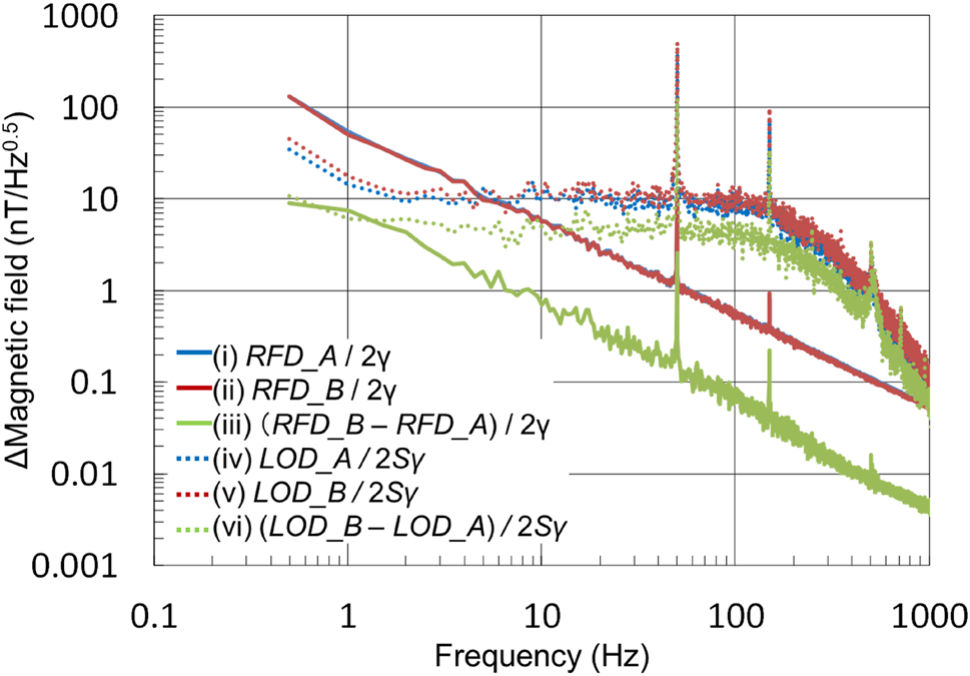
Magnetic field noise spectra without busbar current measured as the resonance frequency difference (*RFD ≡ R*_*H*_ − *R*_*L*_) multiplied by 1/2*γ* and the lock-in-amplifier output difference (*LOD ≡ Y* − *X*) multiplied by 1/2*γS,* where *S* is the ODMR slope at the resonance frequency, *R*_*H*_ and *R*_*L*_, and *γ* the gyromagnetic ratio (28 Hz/nT). (i) *RFD_A* (*RFD* of sensor A)/2*γ* (solid blue line just behind the solid red line), (ii) *RFD_B* (*RFD* of sensor B)/2*γ* (solid red line), (iii) their difference *(RFD_B* − *RFD_A)*/2*γ* (solid green line), (iv) *LOD_A* (*LOD* of sensor A)/2*γS* (dashed blue line), (v) *LOD_B* (*LOD* of sensor *B*)/2*γS* (dashed red line), and (vi) their difference, (*LOD_B* − *LOD_A*)/2*γS* (green dashed line). (iii) is one order of magnitude smaller than (i) or (ii), indicating the effect of external magnetic field noise elimination as common mode. (vi) is also half of (iv) or (v), indicating the effect of excitation light noise elimination as common mode.

First of all, the noise spectra of (i) *RFD_A*/2*γ*, (ii) *RFD_B*/2*γ*, and (iii) (*RFD_B* − *RFD_A*)/2*γ* of the differential detection system was measured without busbar current. Their results are shown in Fig. 3. The spectrum of the difference (iii) is one order of magnitude smaller than those of (i) and (ii), indicating that the differential detection is effective in eliminating external magnetic field noise.

In the same figure, (iv) *LOD_A*/2*γS*, (v) *LOD_B*/2*γS*, and (vi) (*LOD_B* − *LOD_A*)/2*γS* are also shown. (iv)–(vi) are flat at a few nT/Hz^0.5^ from a few to 100 Hz, showing the internal noise floor of the measurement system. The flat floor level of (vi) is ~ 5 nT/Hz^0.5^, which is about half of (iv) or (v), indicating that the differential detection is effective in reducing the common mode noise including the excitation light noise in *LOD*. In order that *RFD* can reflect the effect of high-speed transient current change, the high sensitivity in *LOD* is important.

## 10 mA busbar current detection in a noisy environment

**Figure 4:**
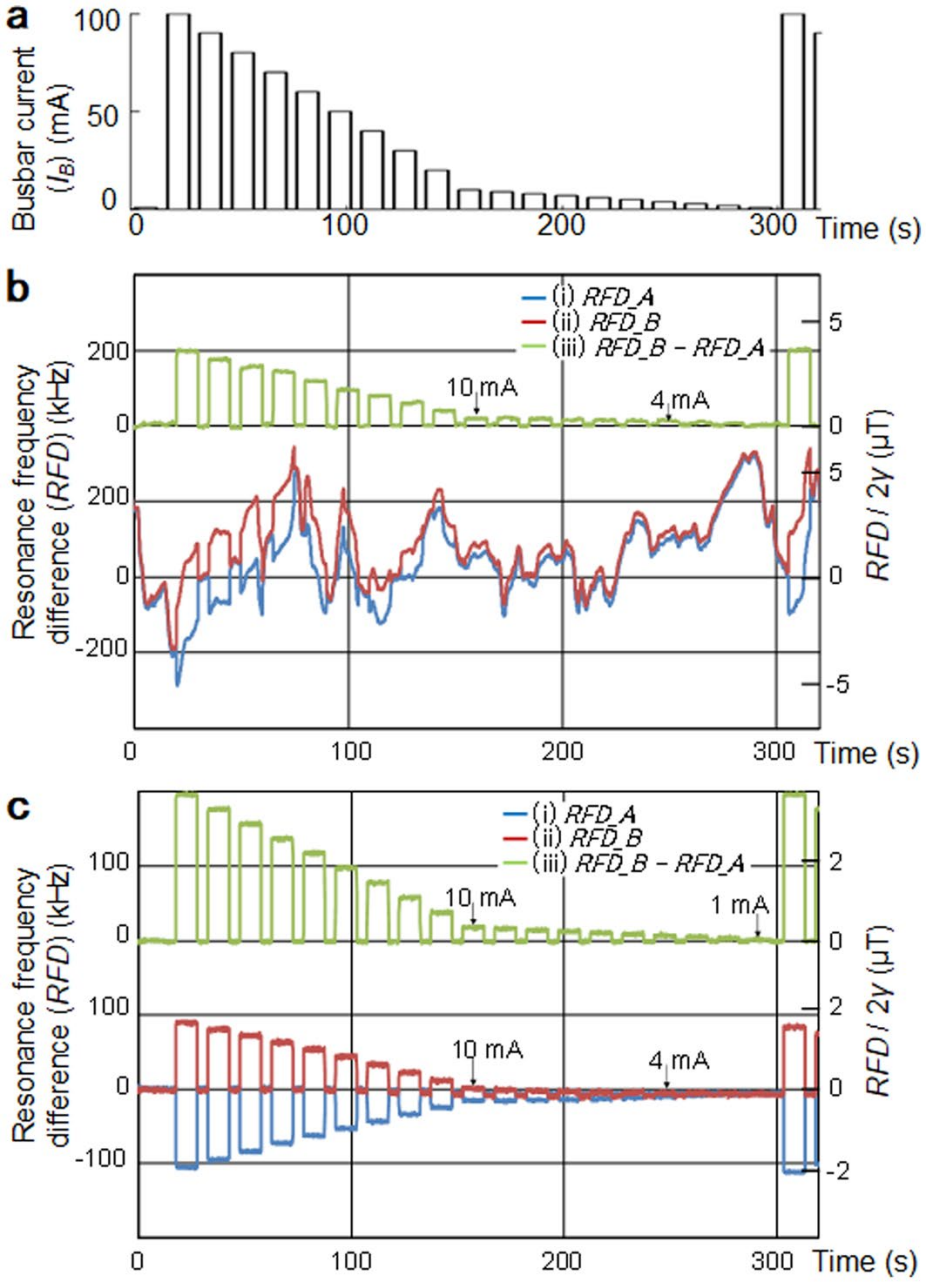
Small current measurement from an external source. (**a**) Pulse train input of 100 mA ~ 1 mA. (**b**) Measured resonance frequency difference (*RFD*), (i) *RFD_A*, (ii) *RFD_B*, and (iii) *RFD_B* − *RFD_A* in the daytime. 10 μT_pp_ external noise in (i) *RFD_A* and (ii) *RFD_B* is eliminated in (iii) *RFD_B* − *RFD_A* and ~ 4 mA is detected in (iii). (**c**) The measurement result at midnight with little external magnetic field noise. ~ 4 mA is detected in (i) *RFD_A* and (ii) *RFD_B*, and ~ 1 mA is detected in (iii) *RFD_B* − *RFD_A*.

Subsequently, a current pulse train from an external current source with the amplitude of 100 mA to 1 mA, as shown in Fig. 4a, is supplied to the busbar to confirm that 10 mA can be detected in the time domain in a noisy environment. Figure 4b shows the obtained waveforms of (i) *RFD_A*, (ii) *RFD_B*, and (iii) (*RFD_B* − *RFD_A*) in the daytime without magnetic shield in the laboratory. The vertical axis on the right side shows the magnetic field scaled with 1/2*γ*. In (i) *RFD_A* and (ii) *RFD_B*, 10 μT_pp_ external noise is observed, but in (iii) (*RFD_B* − *RFD_A*), the external noise is removed, and the current down to ~ 4 mA can be detected. The 10 μTpp external noise is mainly caused by the electric railway in front of our building. Figure 4c shows the measurement result at midnight when the electric railway stops, and a current of 1 mA can be detected.

As shown in Fig. 4c, the *RFD_B* − *RFD_A* for the busbar current of 100 mA was 196 kHz. From this, the magnetic field for 100 mA current was 196/2/2/28 = 1.75 μT and 1.75 μT/100 = 17.5 nT for 1 mA. Since the sensitivity of (vi) (*LOD_B* − *LOD_A*)/2*γS* in Fig. 3 was ~ 5 nT/Hz^0.5^, it is reasonable that 1 mA could be detected in an environment with little noise.

On the other hand, the measured accuracy difference in the daytime and midnight indicates that there certainly is non-common-mode noise that cannot be eliminated by the differential detection. The actual noise level around the battery module in the automobile is estimated to attain 50 μT. We will have to improve the measurement system in Fig. 2e to reduce non-common-mode noise to realize 10 mA accuracy in the actual automobile environment.

## Analog–digital control of the microwave generator frequency for wide dynamic range to measure large current from the battery module

**Figure 5:**
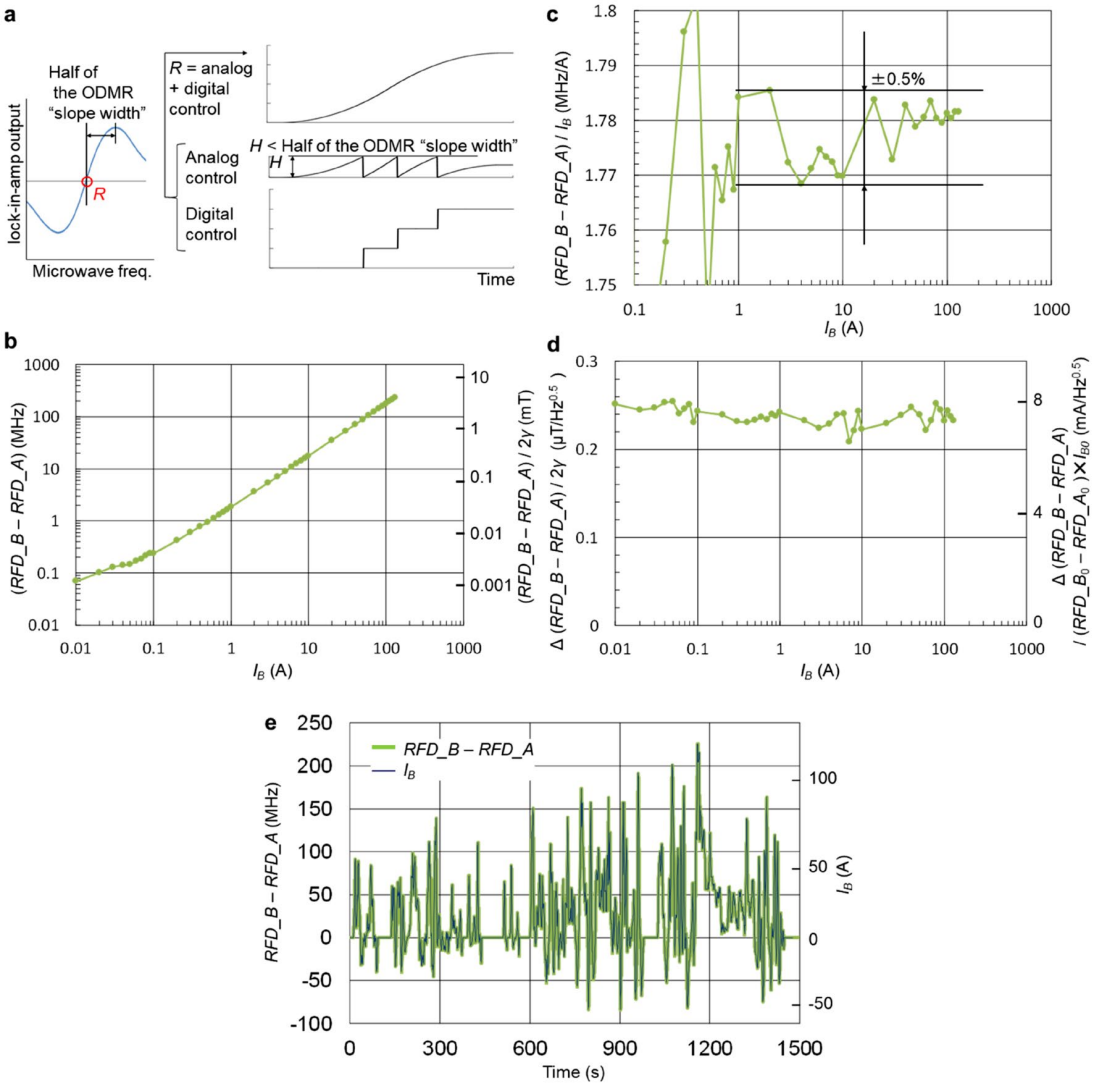
Battery module current measurement. (**a**) Principle of mixed analog–digital control for the microwave generator frequency to trace the resonance frequency over a wide dynamic range. On the left, half of the ODMR “slope width” around the resonance frequency *R* in the spectrum is illustrated. On the right is the example microwave generator frequency control consisting from analog control from the integrator feedback from the lock-in-amplifier and the digital control of the microwave generator center frequency to keep the analog control less than half of the ODMR “slope width” when the magnetic field change is larger than half of the ODMR “slope width”. (**b**) (Resonance frequency difference of sensor B, *RFD*_B)—(that of sensor A, *RFD*_A) when the busbar current of 10 mA to 130 A is supplied from the battery module. Owing to the limited accuracy in the reproducible current source controlling the battery module, the linearity is limited for less than 1 A. (**c**) The ratio of (*RFD_B* − *RFD_A*) to the busbar current as the busbar current changes from 100 mA to 130 A. ± 0.5% linearity is confirmed from 1 to 130 A. (**d**) The fluctuation of (*RFD_B* − *RFD_A*) as the busbar current changes from 10 mA to 130 A. The left vertical axis is the magnetic field scaled with the gyromagnetic ratio 2*γ* = 56 kHz/μT and the right vertical axis is the current scaled with the ratio of (*RFD_B*_*0*_ − *RFD_A*_*0*_)/*I*_*B0*_ (1.78 MHz/A), where (*RFD_B*_*0*_ − *RFD_A*_*0*_) is (*RFD_B* − *RFD_A*) at *I*_*B0*_ = 130 A. The fluctuation is below 0.3 μT/Hz^0.5^ or 8 mA/Hz^0.5^. (**e**) Measured (*RFD_B* − *RFD_A*) in thick green line when WLTC current pattern is supplied to the busbar. Using the ratio 1.78 MHz/A, (*RFD_B* − *RFD_A*) coincided with the whole WLTC pattern of *I*_*B*_ in black line.

The microwave generator can stably trace the resonance frequency using the analog control from the integrator circuit shown in Fig. 2e automatically as long as the resonance frequency change is within a certain limit. This limit is shown in the ODMR spectrum around the resonance frequency illustrated on the left of Fig. 5a as half of the ODMR “slope width”. If (magnetic field change) × *γ* exceeds this limit, additional control is required for the microwave generator frequency to suppress the analog control within the above limit, which is shown on the right of Fig. 5a. The microwave generator center frequency is digitally controlled. If the analog control exceeds a certain value, denoted as *H*, which is less than half of the ODMR "slope width,” the microwave generator center frequency should be digitally adjusted by + *H*. On the other hand, if analog control becomes lower than − *H*, the microwave generator center frequency should be digitally adjusted by − *H*.

Specifically, in the sensor in Fig. 2, half of the ODMR “slope width” was 3.5 MHz. which corresponded to the busbar current of about 7 A. *H* was set as 3 MHz. The microwave generator center frequency received digital control from PC in 100 ms period.

With such a mixed analog–digital control, the busbar current of 10 mA to 130 A, covering the WLTC pattern, was supplied from the battery module in 60 s step. A 8 mm thick busbar was used. The obtained resonance frequency difference is shown in Fig. 5b with the busbar current on the horizontal axis. The left vertical axis of the figure shows the frequency difference in MHz, and the right vertical axis shows the magnetic field converted with 2*γ* = 56 MHz/mT. Owing to the limited accuracy in the reproducible current source controlling the battery module, the linearity is limited for less than 1 A.

Figure 5c shows the ratio of the resonance frequency difference to the busbar current in each step. The variation in the ratio was within ± 0.5% from 1 to 130 A.

Figure 5d shows the magnetic field fluctuation in each step converted from the resonance frequency difference, which were within 0.3 μT/Hz^0.5^ up to 130 A, covering WLTC driving pattern. The vertical axis on the right shows the value converted to the current using the ratio at 130 A (1.78 MHz/A). The current fluctuation was within 8 mA/Hz^0.5^. This 0.3 μT/Hz^0.5^ can be converted to percentage for a better quantification as follows. The *RFD_B* − *RFD_A* for 130 A is 130 × 1.78 = 231 MHz, and the magnetic field is 231/2/2/28 = 2.1 mT, so 0.3 μT/2.1 mT = 0.015%.

Figure 5e shows the measured resonance frequency difference when WLTC current pattern in Fig. 1c is provided. Using the ratio 1.78 MHz/A, the resonance frequency difference traced the whole WLTC current pattern without discernible difference. The integrated state of charge obtained from the resonance frequency difference coincided with that obtained from the WLTC pattern within 0.2%.

The time delay between the microwave generator frequency change and the resonance frequency change should be discussed. Because the microwave generator frequency is controlled so that the integral difference with the resonance frequency becomes zero after a certain time in the order of CR, the microwave generator frequency change has a certain delay from the resonance frequency change. Intermittent digital control of the microwave generator center frequency required for wide dynamic range also increases the delay. However, since the integral difference between the microwave generator frequency and the resonance frequency is zero after a sufficiently long time, their integral values coincide. Because the target is the battery state of charge estimation as the integral value of the resonance frequency difference, as long as the microwave generator frequency can trace the resonance frequency, the integral value of the resonance frequency difference can accurately predict the battery state of charge. For this reason, the fact that the WLTC pattern could be traced by the resonance frequency difference in real time is important.

## ± 1000 A and − 40 °C to + 85 °Coperation

**Figure 6:**
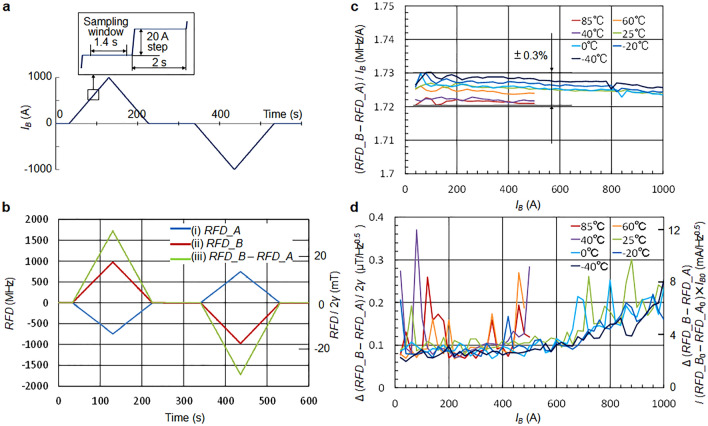
Large current measurement from external source. (**a**) ± 1000 A input current waveform to the busbar stepped by 20 A in each 2 s. The sampling window was cut out from each step, excluding the transient response portion. (**b**) Observed resonance frequency difference (*RFD*), *RFD_A* (blue line), *RFD_B* (red line), and (*RFD_B* − *RFD_A*) (green line). All waveforms are correctly tracing the busbar current. (**c**) The ratio of average value of (*RFD_B* − *RFD_A*) within the sampling window vs. busbar current in each step. ± 0.3% linearity was confirmed in the current range from 40 to 1000 A and the temperature range from − 40 to + 25 °C as well as in the current range from 40 to 500 A and the temperature range from + 40 to + 85 °C. (**d**) Fluctuation of (*RFD_B* − *RFD_A*) in the sampling window of each step. The right vertical axis is the current scaled with the ratio of (*RFD_B*_*0*_ − *RFD_A*_*0*_)/*I*_*B0*_ (1.72 MHz/A), where (*RFD_B*_*0*_ − *RFD_A*_*0*_) is (*RFD_B* − *RFD_A*) at *I*_*B0*_ = 1000 A. Neglecting some noise spikes, which are assumed to originate from the programmable ± 1000 A current supply, the fluctuation is below 0.3 μT/Hz^0.5^ or 10 mA/Hz^0.5^.

Eventually, a busbar current of ± 1000 A stepped at 20 A, as shown in Fig. 6a, was supplied from an external current source. The obtained resonance frequency differences (*RFD*s) are shown in Fig. 6b, which correctly followed the input current. The *RFD* for sensors A and B, *RFD_A* and *RFD_B*, were 740 and 980 MHz respectively at an input current of 1000 A. In the ODMR in Fig. 2d without busbar current, *RFD* was 1050 MHz, indicating that the maximum measurable current should be 1050/980 × 1000 = 1070 (A) in this system.

The sampling window was set as the portion excluding the transient response part of the steps as illustrated in Fig. 6a, and the average and fluctuation of resonance frequency difference were evaluated in the sampling window. Measurements were conducted in the temperature range of − 40 to 85 °C. Owing to the busbar heating, the input current was limited to ± 500 A above 25 °C.

Figure 6c shows the ratio of the resonance frequency difference in average to the busbar current in each step. The variation in the ratio was within ± 0.3%. A subtle temperature dependence can be recognized in this ratio. This is estimated to be caused by the small unbalance of the static magnetic field generated by the neodymium magnet in the temperature bath.

Figure 6d shows the magnetic field fluctuation in each step converted from the resonance frequency difference. Neglecting some noise spikes, which are supposed to originate from the programmable ± 1000A current supply, the fluctuations were within 0.3 μT/Hz^0.5^. The vertical axis on the right shows the value converted to the current using the ratio at 1000 A (1.72 MHz/A). The current fluctuation was within 10 mA/Hz^0.5^.

## Discussions on the accuracy, time-scale responsiveness, and power consumption

The linearity results ± 0.5% in Fig. 5c and ± 0.3% in Fig. 6c were based on the input value to the regenerative battery module current controller and the external current source. However, the accuracy of these devices themselves is specified in % to full scale or rating, which is a few A. Therefore, the true accuracy of the diamond quantum sensors is not expressed in the above figures. The intrinsic accuracy of the diamond quantum sensor is decided by the absolute accuracy of the microwave generator frequency that traces the resonance frequency. The microwave generator (N5172B) used in this experiment has a 40 ppb accuracy, which corresponds to 140 Hz at the resonance frequencies of 2.3 ~ 3.4 GHz and a busbar current value of 0.3 mA. Practically, the accuracy is determined by the fluctuation noise of the ODMR, which was 10 mA/Hz^0.5^, as shown in Figs. 5d and 6d.

In Fig. 6c, a slight temperature dependence within ± 0.3% was observed between − 40 and 85 °C. This temperature dependence was caused by the asymmetry of the static magnetic field applied by the neodymium magnet at the position of sensor A and sensor B. The asymmetry reflects the temperature characteristics of the magnet as the resonance frequency difference *RFD_B* − *RFD_A*. Improving the magnet placement symmetry would remove the temperature dependence.

As for the state of charge estimation accuracy, experimentally verified values in Figs. 5c and 6c are ± 0.5% linearity and ± 0.3% temperature dependence from − 40 to + 85 °C. However, as described above, since the above experimentally verified values are limited by the accuracy of the battery module current controller and the current source, 10 mA/Hz^0.5^ is the theoretical accuracy. From Figs. 5d and 6d, 10 mA/Hz^0.5^ is realized over the entire current and temperature range, 10 mA accuracy is constantly realized. Therefore, we consider that the target of Fig. 1d has been realized.

As for the response time, in Fig. 5a, it took 100 ms cycle time for the PC to detect the analog integrator output by the data acquisition system and then digitally update the center frequency of the microwave generator. As long as the magnetic field change in the 100 ms cycle was less than half of the ODMR “slope width”, which was about 100 μT or 3 MHz × γ, or ~ 7 A busbar current, the microwave generator could trace the resonance frequency and correct state of charge estimation was possible. Usage of dedicated microcontroller instead of PC will reduce the cycle time and enable faster busbar current change.

As for power consumption, each sensor in Fig. 2 received 100 mW excitation light. The fluorescence collection efficiency was ~ 1%^22^. Methods to improve fluorescence collection with lenses^6^ or surface coating^9^ will certainly improve the efficiency, and improving the fluorescence collection efficiency by a factor of N will reduce the excitation light power by 1/N.

Increasing 10% battery usage efficiency reduces 10% battery weight, which will reduce 3.5% running energy and 5% production energy of 20 million new EVs predicted in 2030. These will reduce 1.6 and 12.7 million tons CO_2_, respectively, and 14.3 million tons in total, which corresponds to 0.2% of CO_2_ emission in the 2030 WW transportation field.

## Conclusion

A prototype battery monitor using diamond quantum sensors was developed to estimate the battery state of charge and accurately predict the remaining driving range of EVs. To enable 10 mA current detection in the environmental noise in the automobile, differential detection to eliminate common modes was implemented by placing two diamond quantum sensors on both sides of the busbar. To enable a dynamic range of 1000 A, a mixed analog–digital control of the microwave generator frequency was used to trace the magnetic resonance frequency of the quantum sensor over 1 GHz. An automotive operating temperature range of − 40 ~  + 85 °C was confirmed. The battery module current was measured up to 130 A covering WLTC driving pattern, and the accuracy of the current sensor to estimate battery state of charge was analyzed to be 10 mA, which will almost eliminate the 10% margin currently required and eventually lead to 0.2% CO_2_ reduction emitted in 2030 WW transportation field.

## Methods

### Sensor head structure

The diamond sensors were 2 × 2 × 1 mm^3^ Ib (111) crystals irradiated with a 3 × 10^18^ cm^−2^ electron beam and annealed for 2 h at 1000 °C. Each sensor was adhered to one end of a multimode fiber with a core diameter of 400 μm and NA of 0.5 as shown in Fig. 2a. The NV^−^ concentration was estimated to be in the range of 5–6 ppm. Both the sensor head and the microwave antenna were fixed to the busbar using the ABS (acrylonitrile butadiene styrene) holder as shown in Fig. 2b.

The busbar on which sensors A and B were placed was 2 mm thick and 20 mm wide and made of copper. The [111] NV^−^ axis of the diamond sensor was parallel to the magnetic field generated by the busbar current. The microwave antenna generates a microwave magnetic field perpendicular to the NV^−^ axis. The distance between the busbar surface and the center of the diamond sensor was 5 mm.

The static magnetic field for splitting the ± 1 spin states was applied parallel to the [111] NV^−^ axis by two 30 × 30 × 10 mm^3^ neodymium magnets spaced 90 mm apart, as shown in Fig. 2c. Both sensors A and B can detect the magnetic field in the direction parallel to the busbar surface and perpendicular to the current direction.

### Busbar current-measuring system

In the busbar current measurement system shown in Fig. 2e, 100 mW of excitation light to sensors A and B was supplied from the laser (Coherent Sapphire 532 nm max 300 mW) via a splitter (Thorlabs VA5-PBS251/M). The orthogonal-phase *XY* outputs of one lock-in amplifier were used to control the microwave generator frequencies to trace the low-and high-resonance frequencies *R*_*L*_ and *R*_*H*_ to isolate the temperature drift effect^22^. The microwave generator (Keysight N5172B) has two external FM modulation inputs: Ext1 and Ext2. From Ext1, an FM modulation signal with modulation frequency *F*_*MOD*_ = 2 kHz and deviation depth *F*_*DEV*_ = 3.5 MHz is provided. The modulation frequency, *F*_*MOD*_ = 2 kHz, was chosen to be lower than the typical value (e.g., 18 kHz^1^) because the signal bandwidth required for battery monitoring is not as wide as that for neurobiological measurements^1^, whereas a lower modulation frequency provides a higher contrast in ODMR. The integral output of the lock-in amplifier is provided to Ext2. The integral time constant was CR = 0.1 s. The frequency deviation depth from the 1 V input to Ext2 is denoted by *α*. Because there was a restriction that *F*_*DEV*_ + α ≤ 20 MHz, *α* was chosen to be 16 MHz.

For each sensor, four frequencies generated from the low- and high-resonance frequencies *R*_*L*_ and *R*_*H*_, and their ± *F*_*DEV*_ FM modulation, *R*_*L*_ ± *F*_*DEV*_*,* and *R*_*H*_ ± *F*_*DEV*_, are provided in the non-overlapped timing as shown in Fig. 2f to obtain demodulated ODMR signals in two resonance frequencies as the two orthogonal outputs of one lock-in-amplifier at the same time.

Busbar current was supplied from either external current source directly or via battery module. Keithley 2220 J supplied 100 mA–1 mA in Fig. 4. Regenerative DC current source pCUBE MWBFP3-1008-J supplied 10 mA–130 A (0.2% of full scale accuracy) and WLTC pattern via the battery module AESC Gen4 in Fig. 5. Kikusui PAT20-400 (0.5% of rating accuracy) supplied ± 1000 A in Fig. 6.

### Sensitivity of each sensor

In Eq. (11) in^2^, the sensitivity is defined as the standard deviation of the noise power.

1 $$ \eta = \sqrt {\frac{1}{{T_{trial} }}\mathop \smallint \limits_{0}^{{T_{trial} }} \left[ {B^{meas} \left( t \right)} \right]^{2} dt} \times \frac{1}{{\sqrt {2f_{ENBW} } }} $$

*f*_*ENBW*_ = 5/64 τ_LIA_ is the effective noise bandwidth of the lock-in amplifier, where *τ*_*LIA*_ is the post-filter time constant of the lock-in amplifier.

In Fig.3, using this formula, measurements were taken at *T*_*trial*_ = 2 s and averaged 10 times.

The results showed that (vi) (*LOD_B* − *LOD_A*)/2*Sγ* = 4.7 is certainly smaller than (iv) *LOD_A*/2*Sγ* = 12.8, and (v) *LOD_B*/2*Sγ* = 16.9 nT/Hz^0.5^, and the effect of differential measurement on noise power was also confirmed.

### Magnetic field distribution by busbar current

The 2D magnetic field distribution, assuming a uniform current distribution in the busbar cross-section, was simulated using ANSYS Maxwell. The magnetic field at the sensor position, 5 mm distance from the busbar surface, was 1.5 μT for 100 mA, which almost coincided with 1.7 μT obtained in Fig. 4c.

### Excess heating suppression of busbar

For a battery current measurement up to 130 A in Fig. 5 and a large current measurement of ± 1000 A in Fig. 6, the busbar thickness was increased to 8 mm to suppress busbar heating. The period of the steps was 60 s in Fig. 5, which was shortened to 2 s as shown in Fig. 6a. The 200 s triangular + 1000 A waveform and the -1000 A waveform were applied at 100 s interval.

In the large current measurement, the entire sensor head shown in Fig. 2b and c, including the magnet for the static magnetic field, was installed in a thermostatic bath, and the measurement was carried out at bath temperatures ranging from − 40 to 85 °C. Because the busbar temperature exceeded 100 °C at a bath temperature of 25 °C and a current of 1000 A, the current amplitude had to be limited to ± 500 A above 25 °C to avoid thermal damage to the sensor-head-holder made of ABS. Using the sensor holder material with more heat persistency such as PEEK (PolyEtherEtherKetone), ± 1000 A measurement in + 40 to 85 °C would be possible.

## Data Availability

The data that support the findings of this study are available from the corresponding author upon reasonable request.
